# Association of Matrix Metalloproteinase 9 and Cellular Fibronectin and Outcome in Acute Ischemic Stroke: A Systematic Review and Meta-Analysis

**DOI:** 10.3389/fneur.2020.523506

**Published:** 2020-11-24

**Authors:** Lu Wang, Linghui Deng, Ruozhen Yuan, Junfeng Liu, Yuxiao Li, Ming Liu

**Affiliations:** ^1^Department of Neurology, West China Hospital, Sichuan University, Chengdu, China; ^2^Center of Rehabilitation Medicine, West China Hospital, Sichuan University, Chengdu, China; ^3^National Clinical Research Center for Geriatrics, West China Hospital, Sichuan University, Chengdu, China

**Keywords:** matrix metalloproteinase 9, cellular fibronectin, severe brain edema, hemorrhagic transformation, poor outcome

## Abstract

**Introduction:** The role of matrix metalloproteinase 9 (MMP-9) and cellular fibronectin (c-Fn) in acute ischemic stroke is controversial. We systematically reviewed the literature to investigate the association of circulating MMP-9 and c-Fn levels and MMP-9 rs3918242 polymorphism with the risk of three outcome measures after stroke.

**Methods:** We searched English and Chinese databases to identify eligible studies. Outcomes included severe brain edema, hemorrhagic transformation, and poor outcome (modified Rankin scale score ≥3). We estimated standardized mean differences (SMDs) and pooled odds ratios (ORs) with 95% confidence intervals (CIs).

**Results:** Totally, 28 studies involving 7,239 patients were included in the analysis of circulating MMP-9 and c-Fn levels. Meta-analysis indicated higher levels of MMP-9 in patients with severe brain edema (SMD, 0.76; 95% CI, 0.18–1.35; four studies, 419 patients) and hemorrhagic transformation (SMD, 1.00; 95% CI, 0.41–1.59; 11 studies, 1,709 patients) but not poor outcome (SMD, 0.30; 95% CI, −0.12 to 0.72; four studies, 759 patients). Circulating c-Fn levels were also significantly higher in patients with severe brain edema (SMD, 1.55; 95% CI, 1.18–1.93; four studies, 419 patients), hemorrhagic transformation (SMD, 1.75; 95% CI, 0.72–2.78; four studies, 458 patients), and poor outcome (SMD, 0.46; 95% CI, 0.16–0.76; two studies, 210 patients). Meta-analysis of three studies indicated that the MMP-9 rs3918242 polymorphism may be associated with hemorrhagic transformation susceptibility under the dominant model (TT + CT vs. CC: OR, 0.621; 95% CI, 0.424–0.908; *P* = 0.014). No studies reported the association between MMP-9 rs3918242 polymorphism and brain edema or functional outcome after acute stroke.

**Conclusion:** Our meta-analysis showed that higher MMP-9 levels were seen in stroke patients with severe brain edema and hemorrhagic transformation but not poor outcome. Circulating c-Fn levels appear to be associated with all three outcomes including severe brain edema, hemorrhagic transformation, and poor functional outcome. The C-to-T transition at the MMP-9 rs3918242 gene appears to reduce the risk of hemorrhagic transformation.

## Introduction

Ischemic stroke is one of the most severe neurological disorders and one of the leading causes of disability worldwide (1, 2). The main reasons of deterioration were neurological complications including severe brain edema and hemorrhagic transformation, which may share the common pathophysiological mechanism of blood–brain barrier (BBB) breakdown (3). At present, neuroimaging tests are the main method for diagnosing severe brain edema and hemorrhagic transformation, but these tests are usually performed in the presence of signs of neurological worsening. The neuroimaging tests may delay the effective treatment. Given the limitations of current methods for early detection, new methods are needed to identify these two neurological complications in order to optimize timing of management administration (4).

A change of biomarker levels may precede the appearance of clinical deterioration. Clinical studies have identified that biomarkers of BBB breakdown, such as matrix metalloproteinase 9 (MMP-9) and S100-B, may be associated with clinical deterioration (5–8). Among these biomarkers, MMP-9 has sparked the most interest (9). MMP-9 belongs to a family of zinc-dependent proteolytic enzymes. In animal models, MMP-9 is upregulated in the cerebral ischemic area (10) and degrades the basal lamina around blood vessels in the brain including type IV collagen, fibronectins, and lamina (11–13). As the substrate of MMP-9, fibronectins are located between cell and cell or matrix and consist of cellular fibronectin (c-Fn) and plasma fibronectin (p-Fn). C-Fn is situated nearly exclusively in the endothelium and increases rapidly when vascular damage occurs (14, 15). After stroke onset, high levels of MMP-9 and c-Fn may represent severe damage of the neurovascular unit in injured brain tissue, and when reperfusion begins in the occluded vessels, the disruption of the extracellular matrix may further cause BBB leakage, brain edema, and even hemorrhagic complications in the infarction area (16).

No uniform conclusions have been drawn thus far about associations of circulating MMP-9 levels with the risk of severe brain edema, hemorrhagic transformation, and poor outcome after acute ischemic stroke. Previous systematic reviews suggested a correlation between MMP-9 levels and risk of hemorrhagic transformation (17, 18). While a recent study did not indicate that MMP-9 plasma concentrations were associated with any outcomes including symptomatic intracerebral hemorrhage (sICH), death, and functional outcome (19). Besides, studies focused on c-Fn are very limited, although one study suggested a device that quantified the c-Fn levels was able to stratify patients who developed hemorrhagic transformation (20). In addition, previous studies reported that MMP-9 gene polymorphism, especially the rs3918242 polymorphism (at−1562 locus C/T), regulates expression and thereby influences the levels of circulating MMP-9 (21). Stroke patients with the CT and TT genotypes had significantly higher MMP-9 levels than those with the CC genotypes at the rs3918242 polymorphism (22). MMP-9 rs3918242 polymorphism has already been associated with stroke susceptibility (23–25); however, it is unclear whether or not MMP-9 rs3918242 polymorphism is associated with stroke outcome. Considering these limitations, we aimed to systematically review all the relevant data to investigate (1) whether circulating MMP-9 levels and c-Fn levels might constitute markers of severe brain edema, hemorrhagic transformation, and poor outcome after ischemic stroke; (2) whether variations in the MMP-9 rs3918242 gene were associated with susceptibility to severe brain edema, hemorrhagic transformation, and poor outcome after ischemic stroke.

## Materials and Methods

This meta-analysis was performed and reported according to MOOSE (Meta-analysis of Observational Studies in Epidemiology) guidelines (26).

### Article Search Strategy and Inclusion Criteria

We performed a systematic search for literature published from 1966 to September 2020 without language or other restrictions. The search was conducted in PubMed, Cochrane Library, EMBASE, China National Knowledge Infrastructure, and Wan Fang databases. We used the following keywords and their synonyms in our search strategy: (1) stroke AND (matrix metalloproteinase 9 OR MMP-9 OR cellular fibronectin OR c-Fn) AND (brain edema OR hemorrhagic transformation OR outcome); (2) stroke AND (matrix metalloproteinase 9 OR MMP-9) AND (gene OR polymorphism OR variant OR−1562C/T gene OR rs3918242 polymorphisms). Reference lists from included studies were manually searched for studies relevant to the topic.

No restriction of patient characteristics was applied during the selection of eligible studies. Titles and abstracts were screened for potential eligibility, and then the full texts were reviewed to identify relevant articles that fulfilled the following inclusion criteria: (1) study design was cohort or case-control; (2) any etiology of acute ischemic stroke; (3) patients with and without thrombolysis were included; (4) blood samples were drawn within 48 h after stroke onset and prior to clinical or neuroimaging evidence of any neurological complications and outcome; (5) MMP-9 or c-Fn levels were measured in blood samples; (6) severe brain edema and hemorrhagic transformation were confirmed by neuroimaging including computed tomography (CT) or magnetic resonance imaging (MRI). The first neuroimaging scan was performed within 24 h after admission. Follow-up examination was performed within 14 days after admission or when neurological deterioration occurred; functional outcome was measured by modified Rankin scale (mRS) scores at 3 months after stroke onset; (7) studies on MMP-9 rs3918242 polymorphism reported genotype frequencies in sufficient detail to evaluate the association between MMP-9 polymorphism and outcome; and (8) studies were available as full-text publications. We excluded the studies if they (1) were case reports, letters, review articles, or abstracts and (2) were duplicate studies.

### Diagnostic Criteria

Severe brain edema was defined as brain swelling with a midline shift causing any clinical deterioration (27). Hemorrhagic transformation was defined as the secondary radiographic appearance of hemorrhage after initial neuroimaging had confirmed acute ischemic stroke (5, 13). Hemorrhagic transformation was classified into two types, hemorrhagic infarct (HI) and parenchymal hematoma (PH), according to the European Cooperative Acute Stroke Study criteria. Hemorrhagic transformation was also evaluated as symptomatic or asymptomatic depending on whether the patients suffered clinical deterioration when the hemorrhage occurred. Additionally, sICH was regard as symptomatic hemorrhagic transformation. Detailed definitions have been previously described (13). Poor outcome was defined as mRS score ≥3 at 3-month follow-up.

### Data Collection and Quality Assessment

A standardized data collection form was used to extract the following information from eligible studies: first author, publication year, country of origin, mean age, male gender proportion, time of blood collection, neuroimaging tests and time from stroke onset to the follow-up examination, circulating MMP-9 and c-Fn levels, outcome measures, MMP-9 genotype frequencies, and Hardy–Weinberg equilibrium test results. If the levels of MMP-9 and c-Fn were reported in the form of medians and interquartile ranges (because of skewed distribution), a formula was applied to calculate the mean difference and standard deviation (28). Two reviewers (L.W. and L.D.) independently extracted all data, and any disagreements were resolved between them or in consultation with a third reviewer (Y.L.). Furthermore, we assessed the quality of articles using the Newcastle–Ottawa Scale (NOS) (29). The NOS score consists of the following adapted items: the selection of cohort groups; comparability between 2 groups, and the ascertainment and follow-up of outcome occurrence. NOS score ranges from 0 to 9, with 7 or more indicating high quality.

### Statistical Analysis

We conducted meta-analysis using Stata version 12.0 (STATA Corporation, College Station, TX, USA). When assessing the association between MMP-9 and c-Fn levels and outcomes, we calculated standardized mean differences (SMDs) to describe differences in MMP-9 and c-Fn levels between groups. Pooled odds ratios (ORs) were calculated to determine associations between MMP-9 polymorphism and outcome measures. SMDs and ORs were reported together with the associated 95% confidence intervals (CIs).

We used *I*^2^ to determine heterogeneity across individual studies; *I*^2^ > 50% was considered statistically significant. The random-effects model was adopted when *I*^2^ > 50%; otherwise, the fixed-effects model was used (30). In general, a random-effects meta-analysis model assumes there are various differences in the size of effects observed within the study, whereas a fixed-effects model assumes no variation beyond chance. Thus, when significant heterogeneity exists, the random-effects model allows the effect size to vary from study to study and gives more accurate effect sizes (31).

## Results

### Basic Characteristics of Included Studies

**Figure 1:**
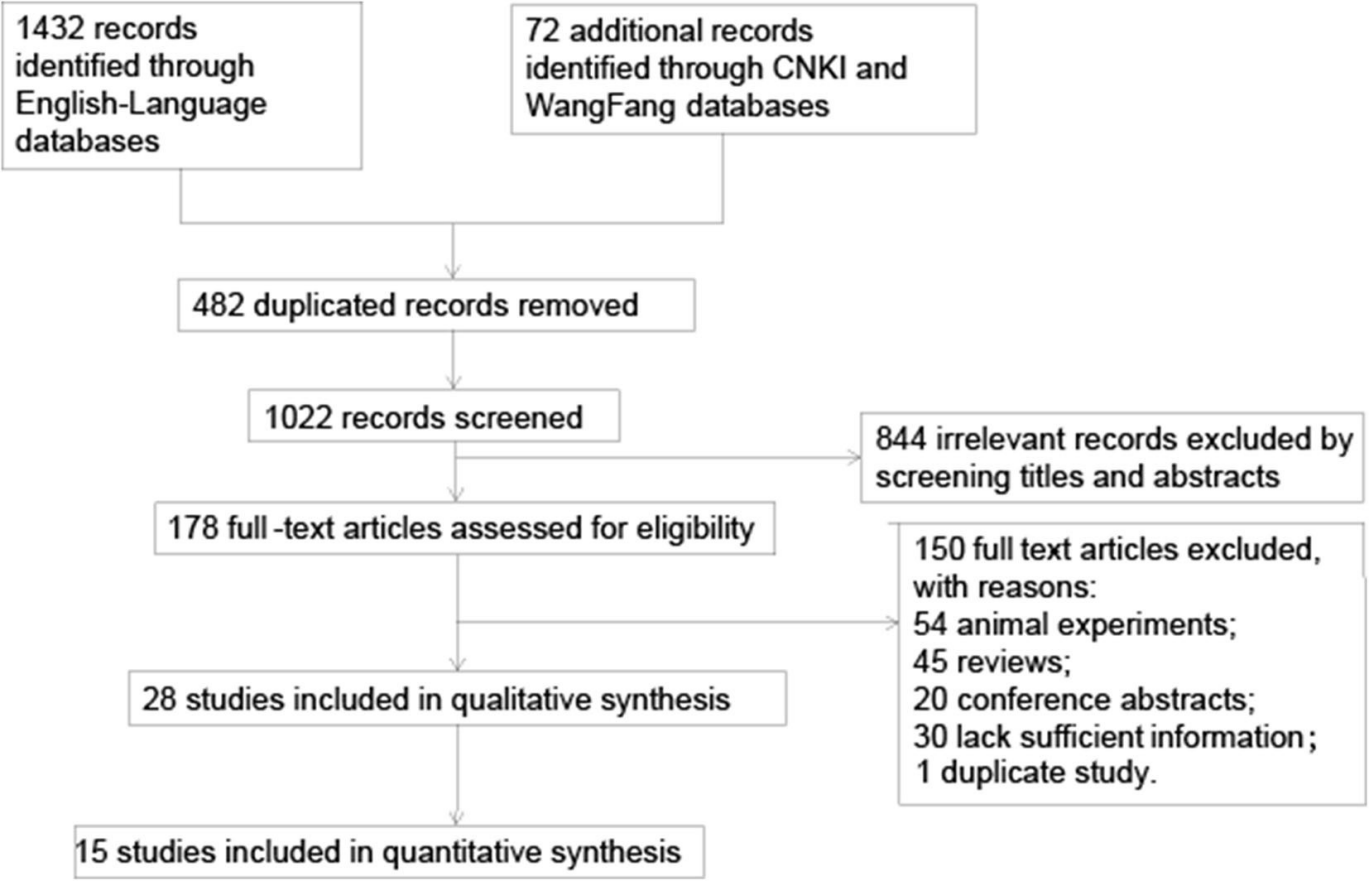
Flowchart of literature search and selection. CNKI, Chinese national knowledge infrastructure; MMP-9, matrix metalloproteinase-9.

The literature selection process is illustrated in Figure 1. We identified 1,504 records after searching five databases. After removing 482 duplicates, 1,022 records were finally selected. Then we retrieved 178 articles and read their full texts after screening the titles and abstracts of 1,022 records. We excluded 54 studies as they were animal experiments; 45 reviews; 20 abstracts with no full text available, whose quality we could not assess; and 30 studies that provided no information about circulating MMP-9 or c-Fn levels or MMP-9 polymorphism. Two articles involved the same participants (9, 32), so we included data only from the more recent publication (32). In the end, 28 articles (6, 8, 15, 19, 27, 32–54) involving 7,239 patients were included in the review (Table 1).

**Table 1:** Main Results of Included Studies of association between matrix metalloproteinase-9 and cellular fibronectin and severe brain edema, hemorrhagic transformation, and poor outcome.

**Studies**	**Markers**	**Type of case (n)**	**Type of control (n)**	**Levels**		
				**Cases**	**Controls**	***P*-value**
9. Millan et al. (32)	MMP-9	BE (15)	NBE (119)	147.2 (54.4, 214.1)	106.4 (69.4, 166)	0.249
12. Rodriguez et al. (27)	MMP-9	BE (10)	NBE (66)	1651.9 ± 591.2	734.4 ± 623.2	<0.001
18. Serena et al. (47)	MMP-9	BE (40)	NBE (35)	150 ± 66	78 ± 59	<0.001
19. Moldes et al. (48)	MMP-9	BE (19)	NBE (115)	131.0 (70.3, 199.9)	108.6 (68.9, 170.5)	0.288
1. Montaner et al. (33)	MMP-9	HT (16)	NHT (23)	135.35 ± 107.2	157.6 ± 126.0	0.050
2. Montaner et al. (34)	MMP-9	HT (15)	NHT (26)	153.1 ± 120.8	126.3 ± 127.5	0.047
3. Castellanos et al. (8)	MMP-9	HT (38)	NHT (212)	193 (163, 213)	62 (40, 93)	<0.001
4. Montaner et al. (35)	MMP-9	HT (9)	NHT (52)	191.4	68.05	0.022
5. Heo et al. (42)	MMP-9	HT (5)	NHT (52)	NA	NA	NA
6. Castellanos et al. (15)	MMP-9	HT (26)	NHT (61)	170.3 (101.4, 96.2)	87.2 (54.8, 115.1)	<0.001
7. Ning et al. (36)	MMP-9	HT (9)	NHT (17)	62.5	38	NA
8. Lucivero et al. (43)	MMP-9	HT (5)	NHT (24)	423.9 ± 185.3		NA
9.Millan et al. (32)	MMP-9	HT (12)	NHT (122)	204.7 (161.6, 235.3)	102 (64.9, 143.5)	<0.001
10. Kazmierski et al. (6)	MMP-9	HT (33)	NHT (425)	730 (499, 1052)	813 (746, 1103)	0.622
11. Leira et al. (37)	MMP-9	HT (55)	NHT (106)	187.3 (100.2, 235.4)	44.2 (23.6, 123.4)	<0.001
12. Rodriguez et al. (27)	MMP-9	HT (8)	NHT (68)	1687.0 ± 428.8	775.9 ± 637.7	<0.001
13. Inzitari et al. (38)	MMP-9	HT (27)	NHT (300)	213.7 (155.2, 483.6)	309.6 (196.4, 491.9)	0.882
14. Jha et al. (39)	MMP-9	HT (29)	NHT (115)	NA	NA	NA
15. Mallolas et al. (40)	MMP-9	HT (49)	NHT (58)	65.3 (23.3, 109.1)	30.3 (21.4, 51.7)	0.029
16. Tsuruoka et al. (41)	MMP-9	HT (14)	NHT (18)	6.82 ± 4.77	>0.05	
16. Tsuruoka et al. (41)	MMP-9	HT (5)	NHT (26)	6.61 ± 3.95	>0.05	
17. Yuan et al. (45)	MMP-9	HT (29)	NHT (139)	244.3 (190.6, 431.4)	110.0 (54.4, 172.2)	<0.001
27. Mechtouff et al. (54)	MMP-9	HT (40)	NHT (108)	NA	NA	NA
28. Maestrini et al. (19)	MMP-9	HT (32)	NHT (223)	NA	NA	NA
7. Ning et al. (36)	MMP-9	PO (NA)	GO (NA)	NA	NA	NA
8. Lucivero et al. (43)	MMP-9	PO (NA)	GO (NA)	NA	NA	NA
9. Millan et al. (32)	MMP-9	PO (73)	GO (61)	129.3 (72.3, 195.3)	92.8 (64.7, 129)	0.021
12. Rodriguez et al. (27)	MMP-9	PO (12)	GO (64)	NA	NA	>0.05
13. Inzitari et al. (38)	MMP-9	PO (115)	GO (212)	292.8 (175.8, 451.5)	312.3 (195.0, 531.3)	0.513
20. Whiteley et al. (49)	MMP-9	PO (112)	GO (156)	872 (619, 1364)	859 (538, 1322)	0.450
21. Abdelnaseer et al. (50)	MMP-9	PO (16)	GO (14)	1111.8 ± 110.36	942.3 ± 143.88	0.003
22. Rodriguez et al. (51)	MMP-9	PO (328)	GO (516)	NA	NA	NA
23. Worthmann et al. (52)	MMP-9	PO (NA)	GO (NA)	NA	NA	NA
24. Zhong et al. (53)	MMP-9	PO (767)	GO (2419)	NA	NA	NA
28. Maestrini et al. (19)	MMP-9	mRS 2-6 (125)	mRS 0-1 (130)	118 (69-194)	120 (75.7–188.2)	0.639
9. Millan et al. (32)	c-Fn	BE (15)	NBE (119)	6.2 (5.4, 7)	3.2 (1.9, 4.4)	<0.001
12. Rodriguez et al. (27)	c-Fn	BE (10)	NBE (66)	41.8 ± 14.8	22.9 ± 14.2	<0.001
18. Serena et al. (47)	c-Fn	BE (40)	NBE (35)	33.7 (25.3, 50.2)	6.7 (4.2, 10.9)	<0.001
19. Moldes et al. (48)	c-Fn	BE (19)	NBE (115)	6.0 (4.1, 6.7)	3.2 (2.1, 4.6)	<0.001
6. Castellanos et al. (15)	c-Fn	HT (26)	NHT (61)	4.8 (3.4, 5.9)	1.7 (1.4, 2.5)	<0.001
9. Millan et al. (32)	c-Fn	HT (12)	NHT (122)	7.9 (6.6, 8.6)	3.2 (1.9, 4.4)	<0.001
11. Leira et al. (37)	c-Fn	HT (55)	NHT (106)	16.3 (5.4, 42.7)	7.1 (2.1, 20.4)	0.001
12. Rodriguez et al. (27)	c-Fn	HT (8)	NHT (68)	47.8 ± 13.6	22.7 ± 13.4	<0.001
9. Millan et al. (32)	c-Fn	PO (73)	GO (61)	3.7 (2.5, 6.2)	3.2 (1.9, 4.2)	0.010
12. Rodriguez et al. (27)	c-Fn	PO (12)	GO (64)	29.7 ± 16.3	21.8 ± 14.3	0.045

*MMP-9, matrix metalloproteinase-9; c-Fn, cellular fibronectin; NA, not available; BE, severe brain edema; NBE, no severe brain edema; HT, hemorrhagic transformation; NHT, no hemorrhagic transformation; PO, poor outcome; mRS 3-6, modified Rankin Scale score from 3 to 6; GO, good outcome; mRS 0-2, mRS, modified Rankin Scale score from 0 to 1; MMP-9, matrix metalloproteinase-9; c-Fn, cellular fibronectin*.

*Baseline MMP-9 and c-Fn Levels are reported as mean ± SD or median (interquartile range)*.

Supplementary Table 1 shows the baseline characteristics of 26 included studies of MMP-9/c-Fn levels and outcome measures. One article was designed as a case-control study (36), and the others were cohort studies. Most studies enrolled patients with all types of acute ischemic stroke, except for three studies including patients without lacunar stroke (33, 34, 40), three studies including patients with cardioembolic stroke involving middle cerebral artery (33–35), and one study including patients with large-vessel occlusion treated with mechanical thrombectomy (54). Treatment with thrombolysis varied across studies: 10 studies included patients treated with thrombolysis (15, 27, 32, 34–36, 38, 42, 48, 54), four studies had some patients who received thrombolysis (19, 39–41), and the remaining 12 studies included patients who did not receive thrombolytic treatment. All studies performed their first CT/MRI scan within 24 h after stroke onset. Besides, almost all studies had their secondary neuroimaging scan within 7 days after onset or when neurological function deteriorated except one study, which had secondary scan done within 14 days (Supplementary Table 1). Nineteen studies were determined to be high quality, and the detailed results of the quality assessment are shown in Supplementary Table 3.

### MMP-9 Levels and Severe Brain Edema

**Figure 2:**
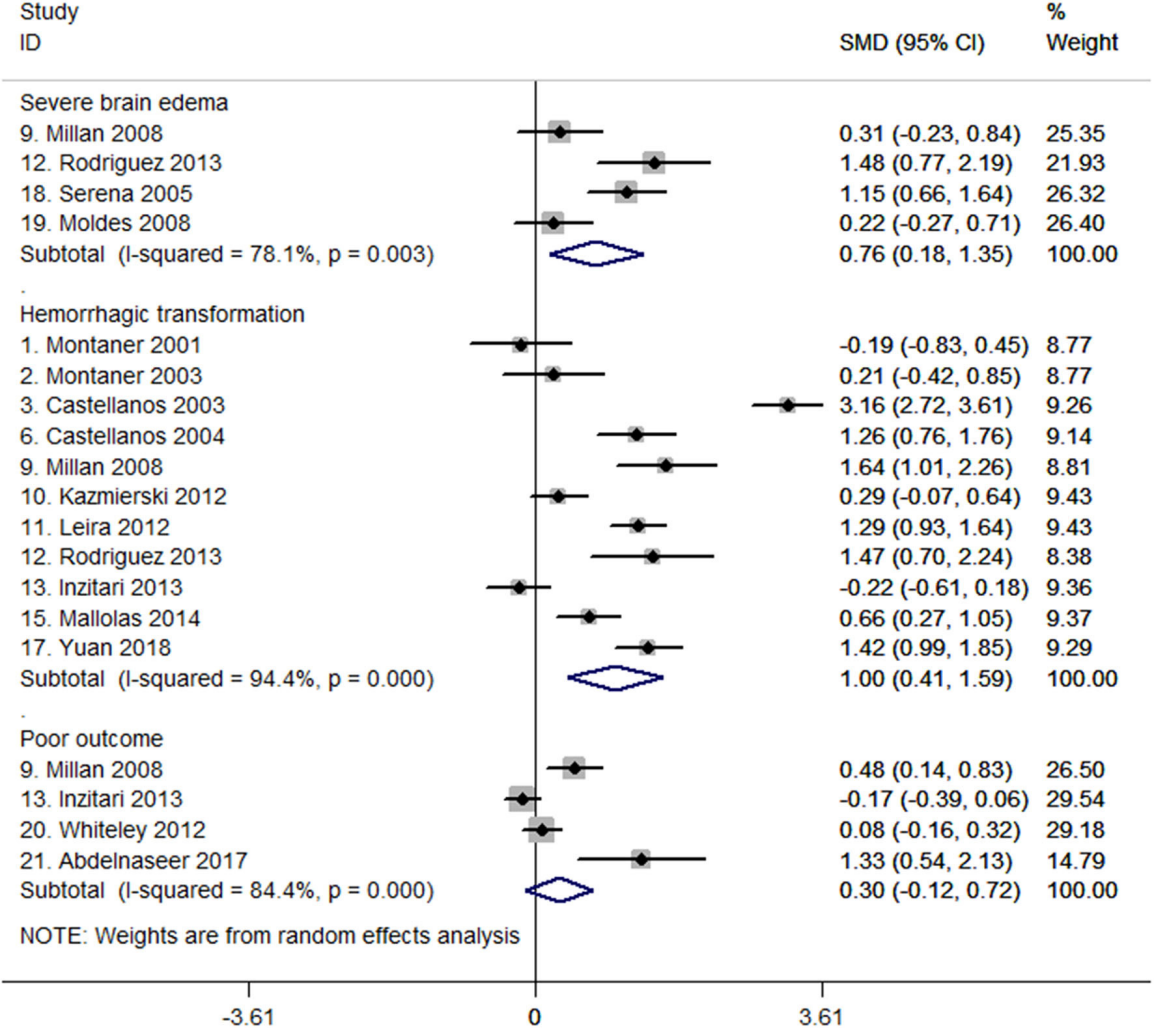
Forest plots of standardized mean difference (SMD) in matrix metalloproteinase-9 (MMP-9) levels between different patient groups.

Four studies (27, 32, 47, 48) suggested that MMP-9 levels were higher in patients with severe brain edema than in those without severe brain edema in the pooled analysis of 419 patients (SMD, 0.76; 95% CI, 0.18–1.35; *P* = 0.01; heterogeneity: *I*^2^ = 78.1%; *P* = 0.003; Figure 2). One study reported MMP-9 per 100 ng/mL increases was independently associated with the risk of severe brain edema in the multivariate analysis (adjusted OR, 1.41; 95% CI, 1.13–1.95) (27).

### MMP-9 Levels and Hemorrhagic Transformation

A total of 19 studies comprising 2,631 patients reported on the relationship between MMP-9 levels and hemorrhagic transformation (6, 8, 15, 19, 27, 32–43, 45, 54). Of the 19 studies, data from 11 studies with 1,709 patients were meta-analyzed to compare MMP-9 levels between patients with or without hemorrhagic transformation (6, 8, 15, 27, 32–34, 37, 38, 40, 45). The forest plot shows that MMP-9 levels were higher in the hemorrhagic transformation group than in the group without hemorrhagic transformation. The SMD between the two groups was 1.00 (95% CI, 0.41–1.59, *P* = 0.001; heterogeneity: *I*^2^ = 94.4%, *P* < 0.001; Figure 2). Among these 11 studies, seven of them reported ORs of association between MMP-9 levels and hemorrhagic transformation (Supplementary Table 2) (8, 15, 33, 34, 37, 40, 45).

For the eight studies not meta-analyzed (19, 35, 36, 39, 41–43, 54), one study reported MMP-9 levels >775 ng/mL were associated with 1.91 times the risk of hemorrhagic transformation (adjusted OR, 2.91; 95% CI, 1.14–7.42) (54); two studies reported that patients with hemorrhagic transformation had higher MMP-9 levels (35, 42); the remaining five studies did not find significant difference in MMP-9 levels between groups with and without hemorrhagic transformation (19, 36, 39, 41, 43). Supplementary Figure 1 also shows that MMP-9 levels were higher in patients with PH than in patients with HI (34, 35, 37, 45), whereas no difference was observed between patients with symptomatic and asymptomatic hemorrhagic transformation (8, 15, 45). Sensitivity analysis showed that systematic exclusion of each study did not significantly affect the pooled results (Supplementary Figure 2).

### MMP-9 Levels and Poor Outcome

Eleven studies with 5,246 patients reported on the association between MMP-9 levels and poor outcome (19, 27, 32, 36, 38, 43, 49–53). Data pooled from four studies with 759 patients (32, 38, 49, 50) showed no difference in baseline MMP-9 levels between groups with poor and good outcome (SMD, 0.30; 95% CI, −0.12 to 0.72; *P* = 0.159; heterogeneity: *I*^2^ = 84.4%, *P* < 0.001; Figure 2). For the seven studies that were not included in statistical pooled analysis, two studies revealed that MMP-9 was correlated with 3-month mRS scores (*r* = 0.508 and 0.508, respectively) (36, 43), and the other 2 studies indicated that MMP-9 was independently associated with 3-month poor outcome (mRS score ≥3) after adjusting for potential confounding factors (ORs, 1.01 and 1.16, respectively) (51, 53). However, the remaining three studies did not find evidence on MMP-9 related to the mRS score at 3 months (19, 27, 52).

### C-Fn Levels and Severe Brain Edema

**Figure 3:**
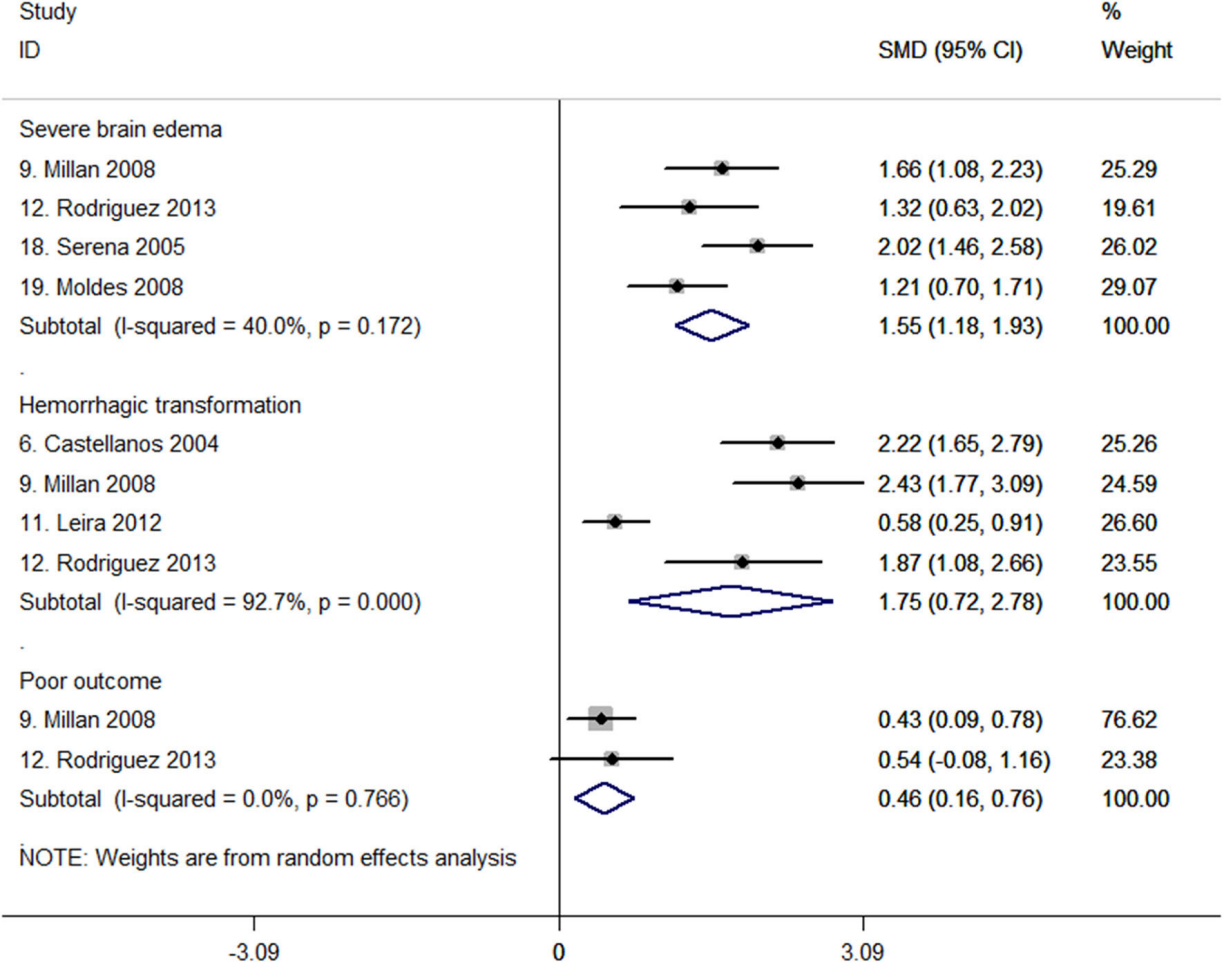
Forest plots of standardized mean difference (SMD) in cellular fibronectin (c-Fn) levels between different patient groups.

Four studies with 419 patients reported the association between c-Fn levels and severe brain edema (27, 32, 47, 48). Meta-analysis of these four studies shows that c-Fn levels were higher in patients with severe brain edema than in those without severe brain edema (SMD, 1.55; 95% CI, 1.18–1.93, *P* < 0.001; heterogeneity: *I*^2^ = 40%; *P* = 0.172, Figure 3). Among them, one study indicated c-Fn per 1-μg increase was independently associated with the risk of severe brain edema (adjusted OR, 1.13; 95% CI, 1.10–1.17) (27).

### C-Fn Levels and Hemorrhagic Transformation

For hemorrhagic transformation, four studies all reported higher c-Fn levels in 458 patients (SMD, 1.75; 95% CI, 0.72–2.78; *P* = 0.001; heterogeneity: *I*^2^ = 92.7%, *P* < 0.001; Figure 3) (15, 27, 37, 48). One study reported that baseline c-Fn level was independently associated with hemorrhagic transformation (adjusted OR, 2.1; 95% CI, 1.3–3.4) after adjusting for age, history of diabetes, baseline National Institutes of Health Stroke Scale score, and MMP-9 levels (15).

### C-Fn Levels and Poor Outcome

Only two studies with 210 patients investigated c-Fn levels in patients with poor and good outcomes (27, 32). One study indicated a significant difference of c-Fn between patients with poor and good functional outcomes in univariate analyses (32). The pooled results suggested c-Fn levels were significantly higher in patients with a poor outcome (SMD, 0.46; 95% CI, 0.16–0.76; *P* = 0.003; heterogeneity: *I*^2^ = 0%; *P* = 0.766; Figure 3). However, no study investigated the independent association between c-Fn levels and poor functional outcome after adjusting for confounding factors.

### MMP-9 Polymorphism and Outcome

**Figure 4:**
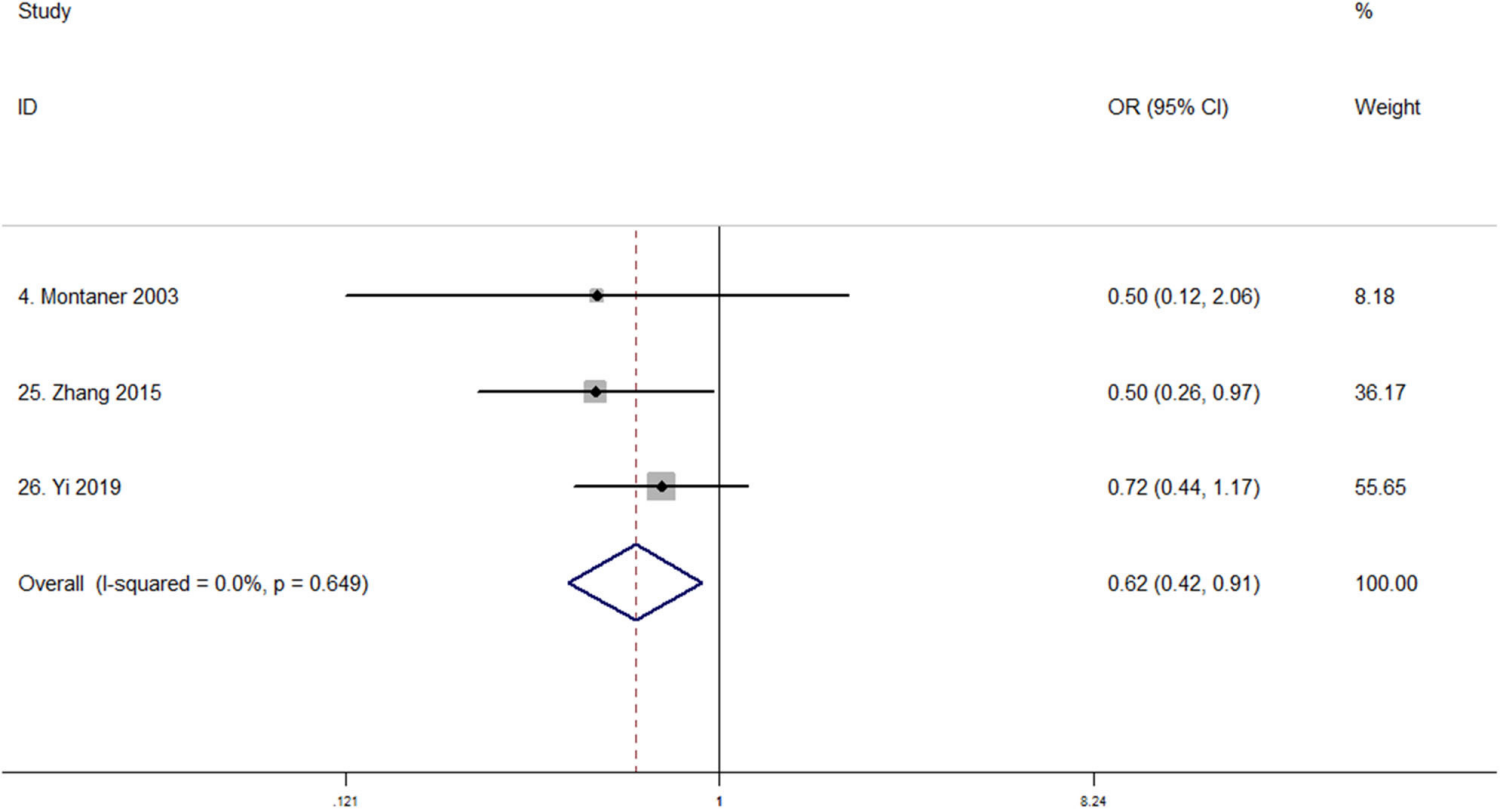
Forest plot of the association between MMP-9 rs3918242 polymorphism and hemorrhagic transformation susceptibility (TT + CT vs. CC). OR, odds ratio. Studies are numbered according to Table 2.

No study reported the relationship between MMP-9 rs3918242 polymorphism and severe brain edema or poor outcome, and only three studies reported MMP-9 rs3918242 polymorphism and hemorrhagic transformation (35, 44, 46). Table 2 displays the baseline characteristics of the three studies that evaluated the potential correlation of MMP-9 rs3918242 polymorphism with the risk of hemorrhagic transformation. Montaner et al. investigated variant rs3918242 and found no association between C-1562T polymorphism and hemorrhagic transformation (35). However, Zhang et al. reported variant rs3918242 was associated with hemorrhagic transformation risk in the Chinese (44), and Yi et al. suggested that the interactions of rs3918242 and rs3787268 in MMP-9 gene may increase hemorrhagic transformation susceptibility (46). In total, 988 patients involving 209 cases and 779 controls (61 Caucasian, 927 Chinese) reported quantitative data. The pooled analysis showed that MMP-9 rs3918242 polymorphism may be significantly associated with hemorrhagic transformation risk under the dominant model (TT + CT vs. CC; OR, 0.621; 95% CI, 0.424–0.908; *P* = 0.014) with no heterogeneity (*I*^2^ = 0%; *P* = 0.649, Figure 4).

**Table 2:** Main results of included studies of MMP-9 rs3918242 polymorphism and HT risk.

**Studies**	**Ethnicity (n)**	**HT/NHT**	**Variants for HT**		**Variants for NHT**		**Follow-up time**	**HWE**	**Conclusions**
			**CT+TT**	**CC**	**CT+TT**	**CC**			
4. Montaner et al. (35)	Caucasian (61)	21/40	3	18	10	30	48 h	YES	C-allele associated with higher rate of HT
25. Zhang et al. (44)	Chinese (222)	84/138	15	69	42	96	14 d	YES	No difference
26. Yi et al. (46)	Chinese (705)	104/601	24	80	177	424	10–14 d	YES	No difference

*NA, not available; HT, hemorrhagic transformation; NHT, non-hemorrhagic transformation; HWE, Hardy–Weinberg equilibrium; CT, computer tomography; SWI, susceptibility-weighted imaging; SNP, single-nucleotide polymorphism; h, hour; d, day*.

## Discussion

To test for the potential association of MMP-9 and c-Fn levels with outcome after stroke, we performed a systematic literature search and meta-analyzed the current literature. We found that MMP-9 and c-Fn levels were elevated in patients with severe brain edema; hemorrhagic transformation and c-Fn were higher in patients with poor outcome. However, MMP-9 levels did not differ significantly between patients with or without poor outcome. Additionally, data from three studies suggested that the T allele at the MMP-9 rs3918242 polymorphism was associated with lower hemorrhagic transformation susceptibility.

The association between MMP-9 and functional outcome was inconsistent. Although a study that we did not include in the pooled analysis revealed the independent association between MMP-9 and poor outcome (53), our study did not find a significant difference of MMP-9 levels between patients with good or poor outcome. A previous study suggested that the different delay of blood sample collection may be one of the possible explanations (19). Our systematic review did not find the discrepancy was due to the time of sample collection, because all studies included had blood samples collected within 48 h after stroke onset, and previous studies reported MMP-9 levels elevate within 2–6 h and remain stable over the first 48 h (19, 54). However, blood samples collected in an early time may have better predictive value for functional outcome. In addition, we did not perform subgroup analysis based on the etiology of stroke and thrombolysis treatment due to limitations of the available data. Whether the discrepancy was due to the etiology of stroke and thrombolysis treatment needs further investigation.

Many studies suggest that MMP-9 and c-Fn participate in the process of brain edema and hemorrhagic transformation (55–57). The currently accepted working model is that stroke-induced injury to vascular endothelium leads to a series of inflammatory responses that trigger the release and activation of MMP-9. Many cells, including neutrophils and endothelial cells, contribute to this increased secretion (58). MMP-9 degrades the extracellular matrix, increases the permeability of the BBB, and destroys the vascular bed. Eventually, collateral circulation or recanalization brings about ischemic tissue reperfusion, leading to brain edema, blood extravasation, and hemorrhagic transformation, coming along with elevated free fragments of c-Fn (5, 58, 59). The c-Fn in basal lamina plays a vital role in the hemostasis by mediating the adhesion of platelets; when degraded by MMP-9, it may lose the normal function and make the BBB disruption more serious (60). Those explanations were also supported by the study of Castellanos in 2007 (9), which found the combination of MMP-9 and c-Fn levels has a better predictive ability for hemorrhagic transformation. One study attributed the severity of hemorrhage to the location of damaged cerebrovascular basal lamina (61). It is possible that the different neurological complications are due at least in part to differences in MMP-9 levels, which remove basal lamina over a smaller or larger area, resulting in severe brain edema, or hemorrhagic transformation. Consistent with this idea, we found higher MMP-9 levels in the more severe hemorrhagic transformation subtype PH than in the subtype HI.

Pooled analysis of three studies suggested that the C-to-T transition in the MMP-9 rs3918242 polymorphism may be associated with a lower risk of hemorrhagic transformation, whereas no study investigated the association between the polymorphic site and the susceptibility of brain edema and poor outcome. It is unclear how the transition affects MMP-9 expression (35, 62), with studies linking the T allele to lower circulating MMP-9 levels (35) or to higher levels (21, 22). Future work should examine whether and how the rs3918242 polymorphism affects MMP-9 levels or activity.

A descriptive review of the association between MMP-9 levels and acute ischemic stroke suggested that circulating MMP-9 level may be a biomarker of large infarction, severe stroke, poor functional outcome, and hemorrhage after thrombolysis (17). Our results are consistent with their study, and we further show that MMP-9 levels and c-Fn levels were both associated with brain edema and hemorrhagic transformation. Additionally, we thoroughly investigated the association of different hemorrhagic transformation subtypes, and the quantitative analysis indicated that MMP-9 may be associated with PH but not symptomatic hemorrhagic transformation.

This systematic review suggests that stroke patients with elevated MMP-9 and/or c-Fn levels are at relatively higher risk of severe brain edema and hemorrhagic transformation, which may facilitate early recognition of patients at high risk of neurological deterioration. This review is the first to our knowledge that brings together MMP-9 and c-Fn levels, MMP-9 gene polymorphism and the risk of brain edema, hemorrhagic transformation, and poor outcome following ischemic stroke. More studies are needed to determine the combined predictive value of MMP-9 and c-Fn for early neurological complications.

At the same time, some limitations in our systematic review should be considered when interpreting the results. First, studies differed in their inclusion/exclusion criteria, treatments, timing of blood sample collection, and timing of follow-up imaging. All these differences may account for the observed heterogeneity, which may reduce the reliability of our analysis. Second, we identified only three studies investigating the potential association between the MMP-9 rs3918242 polymorphism and hemorrhagic transformation susceptibility. Therefore, more work should be performed to determine whether this association exists. Third, we were unsuccessful in our attempts to obtain data on the interval between blood drawing and neuroimaging, and all studies assumed that neuroimaging was performed around the time of neurological worsening occurrence. As a result, we cannot assess whether elevated MMP-9 and c-Fn levels preceded the occurrence of neurological complications or *vice versa*, preventing any conclusions about causality. Indeed, a potential causal relationship between MMP-9 and c-Fn levels and the development of neurological complications and severity remains unclear because of the high heterogeneity in the included studies and other unadjusted confounding factors, including recanalization, glucose levels, blood pressure, and previous treatment with antithrombotic therapy.

## Conclusion

Our meta-analysis showed that higher MMP-9 levels were seen in stroke patients with severe brain edema and hemorrhagic transformation but not poor outcome. Circulating c-Fn levels appear to be associated with all three outcomes including severe brain edema, hemorrhagic transformation, and poor functional outcome. The C-to-T transition at the MMP-9 rs3918242 gene appears to reduce the risk of hemorrhagic transformation. More studies should be performed to investigate the independent association between MMP-9 and c-Fn levels and outcome and to explore the role of rs3918242 polymorphism in neurological complications development.

## Data Availability Statement

All datasets generated for this study are included in the article/Supplementary Material.

## Author Contributions

ML is responsible for the conception and design of the study. LW and LD searched databases and extracted data for systematic review. RY, YL, and JL performed the data analysis. LW, LD, RY, JL, YL, and ML wrote the first draft of the manuscript. LW and JL interpreted the data and wrote the final version. JL and ML revised the draft. All authors critically revised the article for important intellectual content and approved the final version. ML obtained public fundings. All authors contributed to the article and approved the submitted version.

## Conflict of Interest

The authors declare that the research was conducted in the absence of any commercial or financial relationships that could be construed as a potential conflict of interest.

## References

[1] Liu L Wang D Wong KS Wang Y. Stroke and stroke care in China: huge burden, significant workload, and a national priority. Stroke. (2011) 42:3651–4. 10.1161/STROKEAHA.111.635755 22052510

[2] Norrving B Kissela B. The global burden of stroke and need for a continuum of care. Neurology. (2013) 80(3 Suppl. 2):S5–12. 10.1212/WNL.0b013e3182762397 23319486 PMC12443346

[3] Simard JM Kent TA Chen M Tarasov KV Gerzanich V. Brain oedema in focal ischaemia: molecular pathophysiology and theoretical implications. Lancet Neurol. (2007) 6:258–68. 10.1016/S1474-4422(07)70055-8 17303532 PMC2725365

[4] Tsuji K Aoki T Tejima E Arai K Lee SR Atochin DN. Tissue plasminogen activator promotes matrix metalloproteinase-9 upregulation after focal cerebral ischemia. Stroke. (2005) 36:1954–9. 10.1161/01.STR.0000177517.01203.eb 16051896

[5] Álvarez-Sabín J Maisterra O Santamarina E Kase C. Factors influencing haemorrhagic transformation in ischaemic stroke. Lancet Neurol. (2013) 12:689–705. 10.1016/S1474-4422(13)70055-3 23726850

[6] Kazmierski R Michalak S Wencel-Warot A Nowinski WL. Serum tight-junction proteins predict hemorrhagic transformation in ischemic stroke patients. Neurology. (2012) 79:1677–85. 10.1212/WNL.0b013e31826e9a83 22993287

[7] Foerch C Wunderlich MT Dvorak F Humpich M Kahles T Goertler M. Elevated serum S100B levels indicate a higher risk of hemorrhagic transformation after thrombolytic therapy in acute stroke. Stroke. (2007) 38:2491–5. 10.1161/STROKEAHA.106.480111 17673718

[8] Castellanos M Leira R Serena J Pumar JM Lizasoain I Castillo J. Plasma metalloproteinase-9 concentration predicts hemorrhagic transformation in acute ischemic stroke. Stroke. (2003) 34:40–6. 10.1161/01.STR.0000046764.57344.31 12511748

[9] Castellanos M Sobrino T Millan M Garcia M Arenillas J Nombela F. Serum cellular fibronectin and matrix metalloproteinase-9 as screening biomarkers for the prediction of parenchymal hematoma after thrombolytic therapy in acute ischemic stroke: a multicenter confirmatory study. Stroke. (2007) 38:1855–9. 10.1161/STROKEAHA.106.481556 17478737

[10] Rosenberg GA Estrada EY Dencoff JE. Matrix metalloproteinases and TIMPs are associated with blood-brain barrier opening after reperfusion in rat brain. Stroke. (1998) 29:2189–95. 10.1161/01.STR.29.10.2189 9756602

[11] National Institute of Neurological Disorders and Stroke rt-PA Stroke Study Group Tissue plasminogen activator for acute ischemic stroke. N. Engl. J. Med. (1995) 333:1581–7. 10.1056/NEJM199512143332401 7477192

[12] Larrue V von Kummer RR Muller A Bluhmki E. Risk factors for severe hemorrhagic transformation in ischemic stroke patients treated with recombinant tissue plasminogen activator: a secondary analysis of the European-Australasian Acute Stroke Study (ECASS II). Stroke. (2001) 32:438–41. 10.1161/01.STR.32.2.438 11157179

[13] Yaghi S Willey JZ Cucchiara B Goldstein JN Gonzales NR Khatri P. Treatment and outcome of hemorrhagic transformation after intravenous alteplase in acute ischemic stroke: a scientific statement for healthcare professionals from the American heart association/American stroke association. Stroke. (2017) 48:e343–61. 10.1161/STR.0000000000000152 29097489

[14] Murphy PA Hynes RO. Alternative splicing of endothelial fibronectin is induced by disturbed hemodynamics and protects against hemorrhage of the vessel wall. Arterioscler. Thromb. Vasc. Biol. (2014) 34:2042–50. 10.1161/ATVBAHA.114.303879 24903094 PMC4140979

[15] Castellanos M Leira R Serena J Blanco M Pedraza S Castillo J. Plasma cellular-fibronectin concentration predicts hemorrhagic transformation after thrombolytic therapy in acute ischemic stroke. Stroke. (2004) 35:1671–6. 10.1161/01.STR.0000131656.47979.39 15166391

[16] Montaner J Ramiro L Simats A Hernández-Guillamon M Delgado P Bustamante A. Matrix metalloproteinases and ADAMs in stroke. Cell. Mol. Life Sci. (2019) 76:3117–40. 10.1007/s00018-019-03175-5 31165904 PMC11105215

[17] Ramos-Fernandez M Bellolio MF Stead LG. Matrix metalloproteinase-9 as a marker for acute ischemic stroke: a systematic review. J. Stroke Cerebrovasc. Dis. (2011) 20:47–54. 10.1016/j.jstrokecerebrovasdis.2009.10.008 21044610

[18] Wang L Wei C Deng L Wang Z Song M Xiong Y. The accuracy of serum matrix metalloproteinase-9 for predicting hemorrhagic transformation after acute ischemic stroke: a systematic review and meta-analysis. J. Stroke Cerebrovasc. Dis. (2018) 27:1653–65. 10.1016/j.jstrokecerebrovasdis.2018.01.023 29598905

[19] Maestrini I Tagzirt M Gautier S Dupont A Mendyk A-M Susen S. MPO is partially associated with neutrophil deleterious effect in acute cerebral ischemia. Neurology. (2020) 95:e97–108. 10.1212/WNL.0000000000009179 32111692

[20] Fernandes E Sobrino T Martins VC Lopez-Loureiro I Campos F Germano J. Point-of-care quantification of serum cellular fibronectin levels for stratification of ischemic stroke patients. Nanomed. (2020) 30:102287. 10.1016/j.nano.2020.102287 32798732

[21] Blankenberg S Rupprecht HJ Poirier O Bickel C Smieja M Hafner G. Plasma concentrations and genetic variation of matrix metalloproteinase 9 and prognosis of patients with cardiovascular disease. Circulation. (2003) 107:1579–85. 10.1161/01.CIR.0000058700.41738.12 12668489

[22] Li Y Chen L Yao S Chen J Hu W Wang M. Association of polymorphisms of the matrix metalloproteinase 9 gene with ischaemic stroke in a southern chinese population. Cell. Physiol. Biochem. (2018) 49:2188–99. 10.1159/000493823 30257242

[23] He T Wang J Wang XL Deng WS Sun P. Association between the matrix metalloproteinase-9 rs3918242 polymorphism and ischemic stroke susceptibility: a meta-analysis. J. Stroke Cerebrovas. Dis. (2017) 26:1136–43. 10.1016/j.jstrokecerebrovasdis.2016.12.036 28258806

[24] Wang B Wang Y Zhao L. MMP-9 gene rs3918242 polymorphism increases risk of stroke: a meta-analysis. J. Cell. Biochem. (2018) 119:9801–8. 10.1002/jcb.27299 30132967

[25] Wu G Cai H Li G Meng S Huang J Xu H. Influence of the matrix metalloproteinase 9 Geners3918242 polymorphism on development of ischemic stroke: a meta-analysis. World Neurosurg. (2019) 133:e31–61. 10.1016/j.wneu.2019.08.026 31415895

[26] Stroup DF Berlin JA Morton SC Olkin I Williamson GD Rennie D. Meta-analysis of observational studies in epidemiology: a proposal for reporting. JAMA. (2000) 283:2008–12. 10.1001/jama.283.15.2008 10789670

[27] Rodriguez JA Sobrino T Orbe J Purroy A Martinez-Vila E Castillo J. proMetalloproteinase-10 is associated with brain damage and clinical outcome in acute ischemic stroke. J. Thromb. Haemost. (2013) 11:1464–73. 10.1111/jth.12312 23742289

[28] Wan X Wang W Liu J Tong T Estimating the sample mean and standard deviation from the sample size, median, range and/or interquartile range. BMC Med. Res. Methodol. (2014) 14:135 10.1186/1471-2288-14-135 25524443 PMC4383202

[29] Wells G Shea B O'Connell D Peterson J Welch V Losos M The Newcastle-Ottawa Scale (NOS) for Assessing the Quality of Nonrandomised Studies in Meta-Analyses (2011). Available online at: http://www.ohri.ca/programs/clinical_epidemiology/oxford.asp (accessed December 5, 2019).

[30] Higgins JP The Cochrane Collaboration Cochrane Handbook for Systematic Reviews of Interventions Version 5.1.0 [updated March 2011]. (2008). Available online at: www.cochrane-handbook.org. 10.1002/9780470712184 (accessed December 5, 2020).

[31] Smolders B Lemmens R Thijs V. Lipoprotein (a) and stroke: a meta-analysis of observational studies. Stroke. (2007) 38:1959–66. 10.1161/STROKEAHA.106.480657 17478739

[32] Millan M Sobrino T Arenillas JF Rodriguez-Yanez M Garcia M Nombela F. Biological signatures of brain damage associated with high serum ferritin levels in patients with acute ischemic stroke and thrombolytic treatment. Dis. Markers. (2008) 25:181–8. 10.1155/2008/380356 19096131 PMC3827808

[33] Montaner J Alvarez-Sabin J Molina CA Angles A Abilleira S Arenillas J. Matrix metalloproteinase expression is related to hemorrhagic transformation after cardioembolic stroke. Stroke. (2001) 32:2762–7. 10.1161/hs1201.99512 11739970

[34] Montaner J Molina CA Monasterio J Abilleira S Arenillas JF Ribo M. Matrix metalloproteinase-9 pretreatment level predicts intracranial hemorrhagic complications after thrombolysis in human stroke. Circulation. (2003) 107:598–603. 10.1161/01.CIR.0000046451.38849.90 12566373

[35] Montaner J Fernández-Cadenas I Molina CA Monasterio J Arenillas JF Ribó M. Safety profile of tissue plasminogen activator treatment among stroke patients carrying a common polymorphism (C-1562T) in the promoter region of the matrix metalloproteinase-9 gene. Stroke. (2003) 34:2851–5. 10.1161/01.STR.0000098648.54429.1C 14605329

[36] Ning M Furie KL Koroshetz WJ Lee H Barron M Lederer M. Association between tPA therapy and raised early matrix metalloproteinase-9 in acute stroke. Neurology. (2006) 66:1550–5. 10.1212/01.wnl.0000216133.98416.b4 16717217

[37] Leira R Sobrino T Blanco M Campos F Rodriguez-Yanez M Castellanos M. A higher body temperature is associated with haemorrhagic transformation in patients with acute stroke untreated with recombinant tissue-type plasminogen activator (rtPA). Clin. Sci. (2012) 122:113–9. 10.1042/CS20110143 21861843

[38] Inzitari D Giusti B Nencini P Gori AM Nesi M Palumbo V. MMP9 variation after thrombolysis is associated with hemorrhagic transformation of lesion and death. Stroke. (2013) 44:2901–3. 10.1161/STROKEAHA.113.002274 23908067

[39] Jha R Battey TWK Pham L Lorenzano S Furie KL Sheth KN. Fluid-attenuated inversion recovery hyperintensity correlates with matrix metalloproteinase-9 level and hemorrhagic transformation in acute ischemic stroke. Stroke. (2014) 45:1040–5. 10.1161/STROKEAHA.113.004627 24619394 PMC3970574

[40] Mallolas J Rodriguez R Gubern C Camos S Serena J Castellanos M. A Polymorphism in the promoter region of the survivin gene is related to hemorrhagic transformation in patients with acute ischemic stroke. Neuromol. Med. (2014) 16:856–61. 10.1007/s12017-014-8333-7 25344020

[41] Tsuruoka A Atsumi C Mizukami H Imai T Hagiwara Y Hasegawa Y. mmp9 effects of edaravone, a free radical scavenger, on circulating levels of MMP-9 and hemorrhagic transformation in patients with intravenous thrombolysis using low-dose alteplase. J. Stroke Cerebrovas. Dis. (2014) 23:2894–9. 10.1016/j.jstrokecerebrovasdis.2014.07.022 25282183

[42] Heo JH Kim SH Lee KY Kim EH Chu CK Nam JM. Increase in plasma matrix metalloproteinase-9 in acute stroke patients with thrombolysis failure. Stroke. (2003) 34:e48–50. 10.1161/01.STR.0000073788.81170.1C 12750540

[43] Lucivero V Prontera M Mezzapesa DM Petruzzellis M Sancilio M Tinelli A. Different roles of matrix metalloproteinases-2 and−9 after human ischaemic stroke. Neurol. Sci. (2007) 28:165–70. 10.1007/s10072-007-0814-0 17690845

[44] Zhang X Cao X Xu X Li A Xu Y. Correlation between the−1562C/T polymorphism in the matrix metalloproteinase-9 gene and hemorrhagic transformation of ischemic stroke. Exp. Ther. Med. (2015) 9:1043–7. 10.3892/etm.2015.2186 25667675 PMC4316928

[45] Yuan R Tan S Wang D Wu S Cao X Zhang S. Predictive value of plasma matrix metalloproteinase-9 concentrations for spontaneous haemorrhagic transformation in patients with acute ischaemic stroke: a cohort study in Chinese patients. J. Clin. Neurosci. (2018) 58:108–12. 10.1016/j.jocn.2018.09.014 30287248

[46] Yi X Sui G Zhou Q Wang C Lin J Chai Z. Variants in matrix metalloproteinase-9 gene are associated with hemorrhagic transformation in acute ischemic stroke patients with atherothrombosis, small artery disease, and cardioembolic stroke. Brain Behav. (2019) 9:e01294. 10.1002/brb3.1294 31074588 PMC6576165

[47] Serena J Blanco M Castellanos M Silva Y Vivancos J Moro MA. The prediction of malignant cerebral infarction by molecular brain barrier disruption markers. Stroke. (2005) 36:1921–6. 10.1161/01.STR.0000177870.14967.94 16100032

[48] Moldes O Sobrino T Millan M Castellanos M Perez de la Ossa N Leira R. High serum levels of endothelin-1 predict severe cerebral edema in patients with acute ischemic stroke treated with t-PA. Stroke. (2008) 39:2006–10. 10.1161/STROKEAHA.107.495044 18436890

[49] Whiteley W Wardlaw J Dennis M Lowe G Rumley A Sattar N The use of blood biomarkers to predict poor outcome after acute transient ischemic attack or ischemic stroke. Stroke. (2012) 43:86–91. 10.1161/STROKEAHA.111.634089 22020034

[50] Abdelnaseer MM Elfauomy NM Esmail EH Kamal MM Elsawy EH. Matrix metalloproteinase-9 and recovery of acute ischemic stroke. J. Stroke Cerebrovasc. Dis. (2017) 26:733–40. 10.1016/j.jstrokecerebrovasdis.2016.09.043 28063771

[51] Rodriguez-Yanez M Castellanos M Blanco M Garcia MM Nombela F Serena J. New-onset hypertension and inflammatory response/poor outcome in acute ischemic stroke. Neurology. (2006) 67:1973–8. 10.1212/01.wnl.0000247064.53130.91 17159103

[52] Worthmann H Tryc AB Goldbecker A Ma YT Tountopoulou A Hahn A. The temporal profile of inflammatory markers and mediators in blood after acute ischemic stroke differs depending on stroke outcome. Cerebrovas. Dis. (2010) 30:85–92. 10.1159/000314624 20484906

[53] Zhong C Yang J Xu T Xu T Peng Y Wang A. Serum matrix metalloproteinase-9 levels and prognosis of acute ischemic stroke. Neurology. (2017) 89:805–12. 10.1212/WNL.0000000000004257 28747453 PMC5580861

[54] Mechtouff L Bochaton T Paccalet A Crola Da Silva C Buisson M Amaz C. Matrix metalloproteinase-9 relationship with infarct growth and hemorrhagic transformation in the era of thrombectomy. Front. Neurol. (2020) 11:473. 10.3389/fneur.2020.00473 32582006 PMC7296118

[55] Sumii T Lo EH. Involvement of matrix metalloproteinase in thrombolysis-associated hemorrhagic transformation after embolic focal ischemia in rats. Stroke. (2002) 33:831–6. 10.1161/hs0302.104542 11872911

[56] Rosenberg GA Yang Y. Vasogenic edema due to tight junction disruption by matrix metalloproteinases in cerebral ischemia. Neurosurg. Focus. (2007) 22:E4. 10.3171/foc.2007.22.5.5 17613235

[57] Rosell A Cuadrado E Ortega-Aznar A Hernández-Guillamon M Lo E Montaner J. MMP-9-positive neutrophil infiltration is associated to blood-brain barrier breakdown and basal lamina type IV collagen degradation during hemorrhagic transformation after human ischemic stroke. Stroke. (2008) 39:1121–6. 10.1161/STROKEAHA.107.500868 18323498

[58] Turner RJ Sharp FR. Implications of MMP9 for blood brain barrier disruption and hemorrhagic transformation following ischemic stroke. Front. Cell. Neurosci. (2016) 10:56. 10.3389/fncel.2016.00056 26973468 PMC4777722

[59] Dang B Duan X Wang Z He W Chen G. A therapeutic target of cerebral hemorrhagic stroke: matrix metalloproteinase-9. Curr. Drug Targets. (2017) 18:1358–66. 10.2174/1389450118666170427151657 28460607

[60] Brunswick AS Hwang BY Appelboom G Hwang RY Piazza MA Connolly ES Jr. Serum biomarkers of spontaneous intracerebral hemorrhage induced secondary brain injury. J. Neurol. Sci. (2012) 321:1–10. 10.1016/j.jns.2012.06.008 22857988

[61] Hamann GF Okada Y del Zoppo GJ. Hemorrhagic transformation and microvascular integrity during focal cerebral ischemia/reperfusion. J. Cereb. Blood Flow Metab. (1996) 16:1373–8. 10.1097/00004647-199611000-00036 8898714

[62] Ye S. Polymorphism in matrix metalloproteinase gene promoters: implication in regulation of gene expression and susceptibility of various diseases. Matrix Biol. (2000) 19:623–9. 10.1016/S0945-053X(00)00102-5 11102751

