# Supplementary material for: Association of Matrix Metalloproteinase 9 and Cellular Fibronectin and Outcome in Acute Ischemic Stroke: A Systematic Review and Meta-Analysis
Source: Front Neurol. 2020 Nov 24;11:523506. doi: 10.3389/fneur.2020.523506 (PMC7732454; doi:10.3389/fneur.2020.523506)
Supplement: Supplementary file 1 [file Data_Sheet_1.docx]

**Supplemental Table 1:**  Baseline characteristic of included studies of MMP-9/c-Fn levels and severe brain edema, hemorrhagic transformation, and poor outcome.

| First author and year of publication | Country of origin | Sample size | Mean age, yr | % Male | Thrombolysis | Onset to admission (h) | Follow-up examination time | Conclusions | NOS |
| --- | --- | --- | --- | --- | --- | --- | --- | --- | --- |
| 1. Montaner 2001 [33] | Spain | 39 | 74.0 | 51.3 | No | ≤ 12 | 48 h, 5 d and 7 d or when neurologic deterioration | Baseline MMP-9 level predicted late HI. | 7 |
| 2. Montaner 2003 [34] | Spain | 41 | 70.0 | 39.0 | Yes | ≤ 3 | 48 h or when neurologic deterioration | Baseline MMP-9 level predicted HT appearance after t-PA treatment | 6 |
| 3. Castellanos 2003 [8] | Spain | 250 | 72.2 | 53.6 | No | ≤ 24 | 4 d and 7 d or when neurologic deterioration | Baseline plasma MMP-9 concentration was an independent predictor of HT. | 9 |
| 4. Montaner 2003 [35] | Spain | 61 | 71.0 | 44.3 | Yes | ≤ 3 | 48 h or when neurologic deterioration | Baseline MMP-9 level predicted HT appearance after t-PA treatment. | 6 |
| 5. Heo 2003 [42] | South Korea | 57 | NA | NA | Yes | ≤ 4.5 | NA | Hemorrhagic transformations occurred in 5 patients who had higher MMP-9 levels. | 5 |
| 6. Castellanos 2004 [15] | Spain | 87 | 67.4 | 58.6 | Yes | ≤ 6 | 24-36 h | Baseline c-Fn but not MMP-9 was independently associated with HT. | 7 |
| 7. Ning 2006 [36] | USA | 26 | 69.9 | 58.0 | Yes | ≤ 8 | 84% had MRI within 48 h,16% CT within 72 h | MMP-9 levels were not significantly higher in patients with petechial HT than in patients without HT. Hyperacute MMP9 was correlated to poor 3-month modified Rankin Scale outcome (r=0.58, p=0.0005). | 5 |
| 8. Lucivero 2007 [43] | Italy | 29 | 72.0 | 31.0 | No | ≤ 12 | 7 d | No differences were found between baseline MMP-9 levels and HT. MMP-9 values at day seven were positively correlated to mRS score at 3-month follow-up (r=0.508, p=0.037) | 8 |
| 9.Millan 2008 [32] | Spain | 134 | 67.2 | 65.7 | Yes | ≤ 3 | 24-36 h or when neurologic deterioration | Baseline c-Fn and MMP-9 levels both above the predefined cutoff values remained independently associated with HT; c-Fn levels were significantly higher in patients with severe brain edema in univariate analyses; MMP-9 and c-Fn levels were significantly higher in patients with poor outcome in univariate analyses. | 7 |
| 10. Kazmierski 2012 [6] | Poland | 458 | 67.5 | 53.6 | No | ≤ 8 | when neurologic deterioration | Baseline MMP-9 was not independently associated with symptomatic HT. | 6 |
| 11. Leira 2012 [37] | Spain | 161 | NA | NA | No | ≤ 12 | 72 h | Baseline MMP-9 levels and c-Fn levels were independently associated with HT. | 7 |
| 12. Rodriguez 2013 [27] | Spain | 76 | 66.9 | 58.0 | Yes | ≤ 3 | 24-36 h | Baseline c-Fn but not MMP-9 was associated with HT; MMP-9 and c-Fn were independently associated with severe brain edema. | 6 |
| 13. Inzitari 2013 [38] | Italy | 327 | 68.9 | 58.1 | Yes | ≤ 3 | 22-36 h | Baseline MMP-9 was not independently associated with symptomatic HT and 3-month poor outcome. | 6 |
| 14. Jha 2014 [39] | USA | 144 | NA | NA | Some | ≤ 9 | 1.6 ± 1.9 d | MMP-9 was not independently associated with HT. | 4 |
| 15. Mallolas 2014 [40] | Spain | 107 | NA | NA | Part | ≤ 12 | 72±12 h | Baseline serum MMP-9 levels were significantly higher in patients with HT. | 6 |
| 16. Tsuruoka 2014 [41] | Japan | 63 | 74.0 | 56.2 | Some | ≤ 12 | 24 h and 7 d | No significant difference was observed in baseline MMP-9 levels between HT and NHT group. | 7 |
| 17. Yuan 2018 [45] | China | 168 | 67.0 | 53.5 | No | ≤ 24 | 3-14 d when neurologic deterioration | Baseline MMP-9 level independently predicted spontaneous HT. | 8 |
| 18. Serena 2005 [47] | Spain | 75 | 62.2 | 65.3 | No | < 24 | 4 d and 7 d or when neurologic deterioration | Baseline MMP-9 and c-Fn were significantly higher in patients with severe brain edema. | 7 |
| 19. Moldes 2008 [48] | Spain | 134 | 67.5 | 65.7 | Yes | ≤ 3 | 24-36 h | c-Fn levels were significantly higher in patients with severe brain edema in univariate analyses. | 7 |
| 20. Whiteley 2012 [49] | UK | 270 | 74.4 | 41.8 | No | < 24 | NA | Baseline MMP-9 was not independently associated with 3-month poor outcome. | 8 |
| 21. Abdelnaseer 2017 [50] | Egypt | 30 | 61.0 | 50.0 | No | < 24 | NA | There was a statistically significant positive correlation between MMP-9 level on admission and outcome as measured by mRS score after 1-month follow-up after controlling for other variables (P <.001). | 7 |
| 22. Rodrıguez 2006 [51] | Spain | 844 | 70.2 | 41.7 | No | ≤ 24 | NA | Baseline MMP-9 was independently associated with 3-month poor outcome. | 8 |
| 23. Worthmann 2010 [52] | Germany | 69 | 74.0 | 50.7 | NA | ≤ 6 | NA | MMP-9 at 3 days after admission were not independently related to the mRS at 3 months. (β=0.156, p=0.049) | 7 |
| 24. Zhong 2017 [53] | China | 3186 | 62.4 | 63.0 | No | ≤ 48 | NA | Baseline MMP-9 was independently associated with 3-month poor outcome. | 8 |
| 27. Mechtouff 2020 [54] | France | 148 | 69.0 | 60.0 | YES | ≤ 24 | 24 h | MMP-9 at 6 h from admission was independently associated with hemorrhagic transformation. No significant difference was observed in MMP-9 levels at 6 h from admission between poor and good outcome group. | 7 |
| 28. Maestrini 2020 [19] | France | 255 | 70 | 54.9 | Some | ≤ 48 | 6–10 d after admission | MMP-9 was not associated with sICH. MMP-9 was not significantly correlated to mRS score at 3-month (r=-0.04, p=0.518) | 9 |

Abbreviations: MMP-9, matrix metalloproteinase-9; c-Fn, cellular fibronectin NA; NA, not available; CT, computer tomography; MRI, magnetic resonance imaging; h, hour; d, day; w, week; NOS, score on the Newcastle-Ottawa Scale.

**Supplemental Table 2**: Odds Ratios reported in included studies.

| Study no. | Variables of multifactor analysis | Outcome | OR | P-value | Adjustment factors | Univariate analysis |
| --- | --- | --- | --- | --- | --- | --- |
| 12. Rodriguez 2013 [27] | MMP-9 per 100- ng/mL | BE | 1.41 (1.13–1.95) | < 0.01 | age, NIHSS at admission, early CT signs > 33% and hypodensity volume. | 1.41 (1.16–2.19) |
| 1. Montaner 2001 [33] | MMP-9> 144.8 ng/mL | late HI | 9 (1.46, 55.24) | 0.01 | absence of early recanalization，hypertension | 15.2 (1.37-167.98) |
| 2. Montaner 2003 [34] | MMP-9> 191.3 ng/mL | PH | 9.62 (1.31,70.26) | 0.025 | initial NIHSS score, dyslipemia | 8.63 (1.43–51.71) |
| 3. Castellanos 2003 [8] | MMP-9> 140 ng/mL | HT | 16 (3.3,79) | ＜0.001 | body temperature, systolic and diastolic blood pressures, treatment with anticoagulants, presence of early signs of infarction on cranial CT, and ultimate infarct volume or lacunar, stroke subtype | NA |
| 6. Castellanos 2004 [15] | MMP-9 per 10- ng/mL | HT | 1.1 (0.9, 1.3) | 0.086 | age, history of diabetes, baseline NIHSS score, and plasma cellular fibronectin levels | NA |
| 11. Leira 2012 [37] | MMP-9 per 1- ng/mL | HT | 3.7 (1.6, 8.3) | <0.001 | median temperature in the first 24 h, cardioembolic stroke subtype, baseline NIHSS score, diastolic blood pressure, and lesion volume at admission based on diffusion-weighted imaging | NA |
| 14. Jha 2014 [39] | MMP-9 per 1- ng/mL | HT | NA | | | 1.48 (0.58-3.69) |
| 15. Mallolas 2014 [40] | MMP-9 per 1- ng/mL | HT | 1.01(1.00, 1.03) | 0.05 | age, NIHSS at admission, temperature at admission, baseline systolic blood pressure, glucose levels at admission, treatment with rt-PA, lesion volume based on baseline diffusion-weighted imaging, baseline lesion volume based on perfusion-weighted imaging, -241 C/T polymorphism | NA |
| 17. Yuan 2018 [45] | MMP-9> 181.7 ng/mL | HT | 18.8 (6.04, 58.53) | <0.001 | lower platelet count, early CT signs of ischemia, and large infarction area | 20.86 (7.27-59.83) |
| 27. Mechtouff 2020 [54] | MMP-9> 775 ng/mL | HT | 2.91 (1.14-7.42) | 0.03 | sex, glucose level, stroke onset to groin puncture time, IV thrombolysis, and baseline volume  on the DWI-sequence (age, NIHSS score, and systolic blood pressure not retained by the backward selection). | 2.48 (1.16-5.27) |
| 20. Whiteley 2012 [49] | MMP-9 per 1-ng/mL | 3-month mRS≥3 | 1.21 (0.85, 1.73) | 0.28 | NIHSS and age, independence of activities of daily living, prior infection and prescription of statins | 1.11 (0.84 to 1.47) |
| 22. Rodrıguez 2006 [51] | MMP-9 per 1-ng/ mL | 3-month mRS>3 | 1.01 (1.00, 1.01) | < 0.001 | History of diabetes, atrial fibrillation, delay from stroke onset to admission, greater baseline stroke severity, higher body temperature, leukocyte count, serum glucose and fibrinogen levels, blood pressure group, IL-6, TNF-α, ICAM-1, VCAM-1, and stroke subtype | NA |
| 24. Zhong 2017 [53] | log MMP-9 per 1-SD (0.32 ng/mL) | 3-month mRS≥3 | 1.16 (1.06, 1.28) | NA | age, sex, time from onset to randomization, current smoking, alcohol drinking, admission NIHSS score, diastolic blood pressure, plasma glucose, white blood cell counts, use of antihypertensive medications, and history of hypertension, coronary heart disease, and diabetes mellitus. | NA |
| 12. Rodriguez 2013 [27] | c-Fn per 1-µg | BE | 1.13 (1.10–1.17) | < 0.01 | age, NIHSS at admission, early CT signs > 33% and hypodensity volume. | 1.10 (1.04–1.23) |
| 6. Castellanos 2004 [15] | c-Fn per 1-µg | HT | 2.1 (1.3-3.4) | 0.002 | age, history of diabetes, baseline NIHSS score, and MMP-9 levels | NA |

Abbreviations: OR, odds ratio; NA, not available; HT, hemorrhagic transformation; CT, computed tomography; NIHSS, National Institutes of Health Stroke Scale; HI, hemorrhagic infarct; PH, parenchymal hematoma; BE, severe brain edema; mRS, modified Rankin scale score; IV, intravenous thrombolysis; DWI, diffusion-weighted imaging

**Supplemental Table 3:** Quality assessment results of included studies.

|  | Selection | | | | | | |  | Outcome | |  |
| --- | --- | --- | --- | --- | --- | --- | --- | --- | --- | --- | --- |
| Study no. | Representativeness of the exposed cohort | Selection of the non-exposed cohort | Ascertainment of exposure | Outcome not present at start of study | Comparability of cohorts based on the design or analysis | Assessment of outcome | Follow-up long enough for outcome to occur | | | Adequacy of follow-up of cohorts | Total score |
| 1. Montaner 2001 [33] | A (1) | A (1) | A (1) | A (1) | C (0) | A (1) | A (1) | | | A (1) | 7 |
| 2. Montaner 2003 [34] | A (1) | A (1) | A (1) | A (1) | C (0) | A (1) | B (0) | | | A (1) | 6 |
| 3. Castellanos 2003 [8] | A (1) | A (1) | A (1) | A (1) | A (1)B(1) | A (1) | A (1) | | | A (1) | 9 |
| 4. Montaner 2003 [35] | A (1) | A (1) | A (1) | A (1) | C (0) | A (1) | B (0) | | | A (1) | 6 |
| 5. Heo 2003 [42] | A (1) | A (1) | B (0) | A (1) | C (0) | A (1) | B (0) | | | A (1) | 5 |
| 6. Castellanos 2004 [15] | A (1) | A (1) | A (1) | A (1) | A (1) | A (1) | B (0) | | | A (1) | 7 |
| 7. Ning 2006 [36] | A (1) | A (1) | B (0) | A (1) | C (0) | A (1) | B (0) | | | A (1) | 5 |
| 8. Lucivero 2007 [43] | A (1) | A (1) | A (1) | A (1) | B (1) | A (1) | A (1) | | | A (1) | 8 |
| 9. Millan 2008 [32] | A (1) | A (1) | A (1) | A (1) | A (1) | A (1) | B (0) | | | A (1) | 7 |
| 10. Kazmierski 2012 [6] | A (1) | A (1) | A (1) | A (1) | C (0) | A (1) | B (0) | | | A (1) | 6 |
| 11. Leira 2012 [37] | A (1) | A (1) | A (1) | A (1) | B (1) | A (1) | B (0) | | | A (1) | 7 |
| 12. Rodriguez 2013 [27] | A (1) | A (1) | A (1) | A (1) | C (0) | A (1) | B (0) | | | A (1) | 6 |
| 13. Inzitari 2013 [38] | A (1) | A (1) | A (1) | A (1) | C (0) | A (1) | B (0) | | | A (1) | 6 |
| 14. Jha 2014 [39] | D (0) | C (0) | A (1) | A (1) | C (0) | A (1) | B (0) | | | A (1) | 4 |
| 15. Mallolas 2014 [40] | A (1) | A (1) | A (1) | A (1) | C (0) | A (1) | B (0) | | | A (1) | 6 |
| 16. Tsuruoka 2014 [41] | A (1) | A (1) | A (1) | A (1) | C (0) | A (1) | A (1) | | | A (1) | 7 |
| 17. Yuan 2018 [45] | A (1) | A (1) | A (1) | A (1) | A (1) | A (1) | A (1) | | | A (1) | 8 |
| 18. Serena 2005 [47] | A (1) | A (1) | A (1) | A (1) | C (0) | A (1) | A (1) | | | A (1) | 7 |
| 19. Moldes 2008 [48] | A (1) | A (1) | A (1) | A (1) | C (0) | A (1) | A (1) | | | A (1) | 7 |
| 20. Whiteley 2012 [49] | A (1) | A (1) | A (1) | A (1) | A (1) | A (1) | A (1) | | | A (1) | 8 |
| 21. Abdelnaseer 2017 [50] | A (1) | A (1) | A (1) | A (1) | A (1) | A (1) | B (0) | | | A (1) | 7 |
| 22. Rodrıguez 2006 [51] | A (1) | A (1) | A (1) | A (1) | A (1) | A (1) | A (1) | | | A (1) | 8 |
| 23. Worthmann 2010 [52] | A (1) | A (1) | A (1) | A (1) | C (0) | A (1) | A (1) | | | A (1) | 7 |
| 24. Zhong 2017 [53] | A (1) | A (1) | A (1) | A (1) | A (1) | A (1) | A (1) | | | A (1) | 8 |
| 25. Zhang 2015 [44] | A (1) | A (1) | A (1) | A (1) | C (0) | A (1) | A (1) | | | A (1) | 7 |
| 26. Yi 2019 [46] | A (1) | A (1) | A (1) | A (1) | C (0) | A (1) | A (1) | | | A (1) | 7 |
| 27. Mechtouff 2020 [54] | A (1) | A (1) | A (1) | A (1) | C (0) | A (1) | A (1) | | | A (1) | 7 |
| 28. Maestrini 2020 [19] | A (1) | A (1) | A (1) | A (1) | A (1)B(1) | A (1) | A (1) | | | A (1) | 9 |


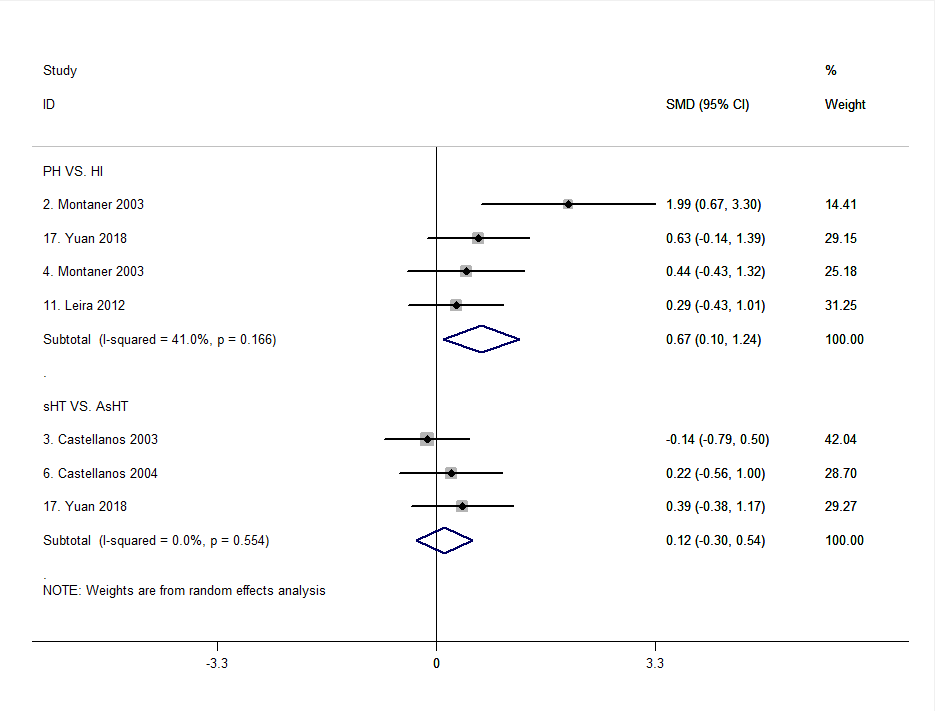


**Supplemental Figure 1**: Standardized mean difference (SMD) in MMP-9 levels between patient with and without different subtypes of hemorrhagic transformation.

Abbreviations: SMD: Standardized Mean Difference; PH, parenchymal hemorrhage; HI, hemorrhagic infarct; sHT, symptomatic HT; AsHT, asymptomatic HT.

**Supplemental Figure 2** Funnel plot of standardized mean difference (SMD) in baseline MMP-9 levels between patient with and without hemorrhagic transformation.

**Supplemental Figure 3** Sensitivity analysis of standardized mean difference (SMD) in baseline MMP-9 levels between patient with and without hemorrhagic transformation.
